# Supplementary material for: Cannabidiol (CBD) Protects Lung Endothelial Cells from Irradiation-Induced Oxidative Stress and Inflammation In Vitro and In Vivo
Source: Cancers (Basel). 2024 Oct 24;16(21):3589. doi: 10.3390/cancers16213589 (PMC11544820; doi:10.3390/cancers16213589)
Supplement: Supplementary file 1 [file cancers-16-03589-s001.zip › cancers-3248231-supplementary.pdf]

cancers

MDPI

Supplementary Materials: Cannabidiol (CBD) Protects Lung Endothelial Cells from Irradiation-Induced Oxidative Stress and Inflammation In Vitro and In Vivo

Lisa Bauer, Bayan Alkotub, Markus Ballman, Morteza Hasanzadeh Kafshgari, Gerhard Rammes and Gabriele Multhoff

Table S1. Individual data points of graphs shown in Figure 1.

**Fig. 1A-C ROS in H5V**		fold change, normalized to control at t0 (pre-irradiation)			
0Gy (control)	t0	1	1	1	1
	t1	2.492953	2.38	2.273682	2.350626
	t2	3.5097	3.43	3.228057	3.039537
0Gy NAC	t0	0.919382	1.543818	1.143865	0.724424
	t1	1.232334	2.361965	1.572503	0.905901
	t2	1.691546	2.96365	1.961111	0.98156
0Gy CBD	t0	1.09876	0.957137	0.871407	
	t1	1.954207	1.910328	1.201126	
	t2	2.523662	2.676485	1.291031	
0Gy NAC + CBD	t0	0.833077	1.390063	0.914651	0.915865
	t1	1.078934	1.834106	1.18768	1.18647
	t2	1.425491	2.214281	1.387208	1.257455
4Gy	t1	3.187219	3.613148	2.815378	3.0851
	t2	5.077068	5.874269	4.298343	4.805336
4Gy + CBD	t1	1.665962	1.876596	2.385724	
	t2	2.163079	3.034498	4.051357	
4Gy + NAC	t1	2.527459	2.764004	2.256409	
	t2	3.411879	3.380832	2.911377	
4Gy + NAC + CBD	t1	2.055129	2.398596	1.67418	2.089612
	t2	2.748396	2.661794	2.028834	2.244193
6Gy	t1	4.275709	3.952964	2.84084	3.5075
	t2	6.104782	6.3236	3.966026	5.417296
6Gy + CBD	t1	1.740059	2.426076	2.744465	1.959057
	t2	2.174294	3.578037	4.680119	2.558463
6Gy + NAC	t1	2.713097	2.081145	1.954796	
	t2	3.682199	2.46025	2.4989	
6Gy + NAC + CBD	t1	2.176869	1.778925	2.396032	
	t2	2.933634	1.845099	2.858667	
**Fig. 1D ROS in primary luEC in vitro**					
0Gy	t0	1	1	1	
	t1	2.86	1.29	1.85	
	t2	3.73	1.69	2.53	
0Gy + CBD	t0	0.81	0.72	0.64	
	t1	1.88	0.93	1.21	
	t2	2.59	1.07	1.35	
4Gy	t0	1	1	1	
	t1	3.26	1.61	3.23	
	t2	4.05	3.89	5.48	
4Gy + CBD	t0	0.78	0.96	0.91	
	t1	1.94	1.7	2.47	
	t2	2.26	2.3	3.96	

Table S2. Individual data points of graphs shown in figure 2.

**Fig. 2A yH2AX in H5V**				
normalized to ß-actin	ctrl	CBD 10µM	4Gy	4Gy + CBD
	0.08696387	0.10616041	0.85395986	0.68732493
	0.56279	0.362684	0.974684	0.458273
	0.019507	0.016978	0.823102	0.733936
	0.66647	0.783243	1.076776	0.755359
**Fig. 2B-C Apoptosis in H5V**				
		Alive		
control	85.3	88.4	87.1	
CBD 10µM	87.5	88.1	87.8	
4Gy	71.6	71.7	72.6	
CBD 10µM + 4Gy	77.6	77.3	77.7	
		Necrotic		
control	4.51	4.39	4.21	
CBD 10µM	3.65	4.28	4.66	
4Gy	6.02	7.85	7.65	
CBD 10µM + 4Gy	6.25	6.69	6.22	
		Early Apoptosis		
control	3.41	2.69	3.15	
CBD 10µM	4.64	2.07	2.06	
4Gy	12.6	14.1	13.6	
CBD 10µM + 4Gy	10.4	8.02	8.11	
		Late Apoptosis		
control	6.77	4.51	5.54	
CBD 10µM	4.21	5.6	5.5	
4Gy	9.7	6.38	6.14	
CBD 10µM + 4Gy	5.76	8.02	7.94	
**Fig. 2D Cell cycle distribution in H5V**				
			G1-phase	
Ctrl		51.2	51.7	52.5
CBD		65.5	56.7	60.7
			S-phase	
ctrl		32.2	31.2	31.5
CBD		18.5	30.5	30.2
			G2-phase	
ctrl		14.1	16.7	16.1
CBD		14.7	12.8	9.13
**Fig. 2E-F Apoptosis in luECs**				
		Alive		
ctrl		40.3	43	
16Gy		36.1	37.1	
16Gy + CBD		47.2	46.9	
		Necrotic		
ctrl		2.95	2.13	
16Gy		2.36	2.96	
16Gy + CBD		1.33	3.7	
		Early apoptosis		
ctrl		30.6	36.8	
16Gy		42.8	39.7	
16Gy + CBD		33.7	29.2	
		Late apoptosis		
ctrl		26.2	18	
16Gy		18.7	20.3	
16Gy + CBD		17.7	20.2	

Table S3. Individual data points of graphs shown in Figure 3.

**Fig. 3A HO-1 in H5V**	ctrl	10µM CBD	4Gy	4Gy + CBD
normalized to ß-actin, then to control	1	3.195032	1.722989	3.16774
	1	2.488107	1.544943	2.264293
	1	2.403741	1.188776	5.916877
	1	2.068048		
**Fig. 3B HO-1 in lung**	control	16Gy	16Gy + CBD 20mg/kg	
normalized to ß-actin, then to control	1	1.05348	2.506101	
	1	0.99464	2.331856	
	1	0.997383	1.978858	
	1	1.198557	1.978327	

Table S4. Individual data points of graphs shown in Figure 4.

**Fig. 4 A-D**		2 weeks			10 weeks		
**Inflammatory markers**		ctrl	16Gy	16Gy + CBD	ctrl	16Gy	16Gy + CBD
ICAM-1	percentage	89.8	95.4	87.5	92.8	90.1	91.8
		92	93.2	90.5	93.8	92.5	88.3
		92.9	91.7	91.6	93.2	92.2	90.5
		91.8	93.8	90.3	93.4	90	
	MFI	20.4	27.8	10.6	24.1	19.2	21.8
		21.7	21.9	17.9	27.2	21	22.4
		19.6	27.9	21.4	22.8	26.4	12.5
		17.9	18.9	16.8	16.5		
		ctrl	16Gy	16Gy + CBD	ctrl	16Gy	16Gy + CBD
ICAM-2	percentage	88.4	91.5	92.4	96.6	94.1	93.5
		92.8	95.3	93	91	96.8	87.1
		93	96.1	92.6	94.6	95.1	91.3
		95	94.8	92.9	95.4	99.5	
	MFI	8.82	13.7	12.5	13.6	19.1	14
		8.62	11.1	13.9	9.54	18	11.6
		10.2	15.1	15.9	14.4	18	8.06
		10.1	20.2	11.5	13.1	27	
		ctrl	16Gy	16Gy + CBD	ctrl	16Gy	16Gy + CBD
VCAM-1	percentage	16.3	16.9	15.6	20.4	13.2	21.6
		20.5	17.5	15.2	17	12.9	21.6
		14.5	23.4	22.4	16.1	13.5	10.2
		11.3	9.23	13.6	16	12.3	
	MFI	2.35	2.73	2.65	3.13	2.73	2.8
		2.27	2.81	2.46	2.73	2.54	2.24
		2.38	2.45	2.88	2.73	2.5	2.65
		2.31	3.03	2.74	3.13	2.27	
		ctrl	16Gy	16Gy + CBD	ctrl	16Gy	16Gy + CBD
MCAM	percentage	86.7	96.1	82.7	89.7	87	91.6
		89.8	93.9	85.6	91.7	90.1	89.8
		94.4	92	92.6	90.5	90.2	89.7
		92.9	94.2	90.3	92.2	89	
	MFI	12.4	21	6.79	11.1	9.6	11.4
		11.3	19.4	9.87	12.8	11.1	7.79
		12.1	15.9	11.8	12.2	12.3	13.9
		9.73	10.8	9.65	10.9	8.9	

Table S5. Individual data points of graphs shown in Figure 5.

**Fig. 5A-B**		2 weeks			10 weeks		
**Angiogenic markers**		ctrl	16Gy	16Gy + CBD	ctrl	16Gy	16Gy + CBD
VE-Cadherin	percentage	76.7	90.9	71.6	90.1	82.4	86
		83.5	88.1	78.3	75.5	87.3	83.7
		81.8	88.6	80.4	89.3	87.5	81.9
		87.6	91.8	84.2	89.5	87.6	
	MFI	7.23	9.33	6.81	11.7	8.99	10.4
		5.76	9.93	5.44	5.51	10.4	8.95
		7.16	10.6	7.03	11.6	10.3	7.43
		7.36	12.5	7.04	10.8	10.7	
		ctrl	16Gy	16Gy + CBD	ctrl	16Gy	16Gy + CBD
Endoglin	percentage	69.6	82.6	85.1	88.4	85	87.4
		66	79.4	82.3	83.3	88.1	80.3
		80.8	89.1	87.2	86.4	86.7	62.2
		76.1	90.9	84.2	86.5	92.1	
	MFI	3.98	5.23	4.26	5.42	6.78	3.32
		3.39	4.67	5.33	5.33	7.27	5.53
		4.61	6.07	7.14	5.84	10.8	5.57
		4.49	5.33	4.48	5.46	14	

Table S6. Individual data points of graphs shown in Appendix.

**Appendix Fig. A2 a**	ROS in H5V after 24h		
	normalized to control		
control	CBD 10µM	4Gy	4Gy + CBD 10µM
1	0.53956835	1.04316547	0.76258993
1	0.80877193	1.35964912	0.99122807
1	0.71816284	1.03966597	0.61169102
1	0.89692102	1.99464525	1.2021419
1	0.88888889	1.77777778	0.83492063
**Appendix Fig. A2 b**	Clonogenic survival in H5V		
	normalized to control		
ctrl	CBD 10µM	4Gy	4Gy + CBD 10µM
1	0.77777778	0.42735043	0.47863248
1	1.09278351	0.28865979	0.40206186
1	0.67826087	0.46086957	0.37391304
1	0.95454545	0.22727273	0.81818182
**Appendix Fig. A4 b**	Leukocyte adhesion to luECs		
	16 Gy	16 Gy + CBD 20 mg/kg	
	491	283	
	260	178	
	3074	167	
	2622	67	

**Appendix Fig. A5 a-b**	2 weeks			10 weeks		
CD34	ctrl	16Gy	16Gy + CBD	ctrl	16Gy	16Gy + CBD
percentage	29	35	37.4	67.2	43.9	73.8
	32.9	55.6	57	63.6	45.6	73.9
	34	53.5	51.6	63.8	53.7	69.1
	39	46.8	46.3	56.1	59.4	
MFI	2.82	2.75	3.22	5.41	3.79	7.33

	2.45	2.8	3.28	4.98	3.7	5.67
	2.97	4.32	4.61	5.12	4.22	7.56
	2.8	4.29	3.82	4.44	4.39	
Integrin-beta-3	ctrl	16Gy	16Gy + CBD	ctrl	16Gy	16Gy + CBD
percentage	31.2	32.5	22.4	32.8	27.9	35.5
	28.8	32	28.7	29.7	41.4	32.2
	26.6	22.9	16.8	37.3	31	29.5
	23	19.8	25.9	29.9	29	
MFI	3.6	3.5	3.9	4.31	3.62	3.89
	3.8	4.48	3.43	4.87	6.33	3.92
	3.62	3.74	3.43	5.05	4.1	3.77
	3.58	4.31	3.74	5.14	3.99	

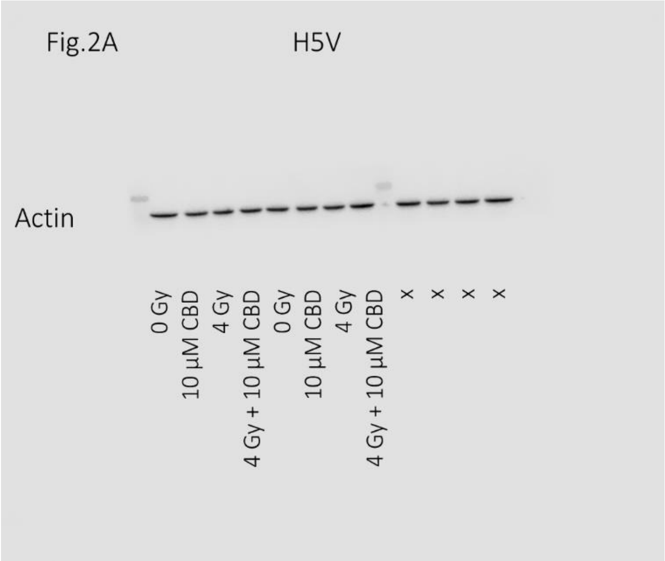

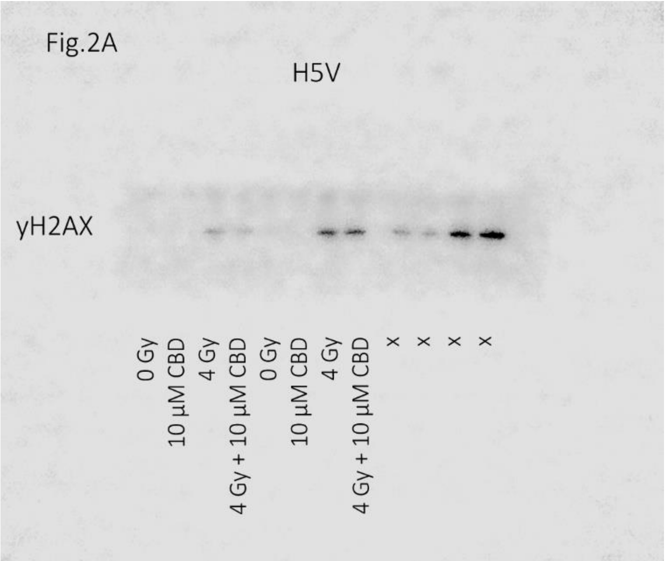

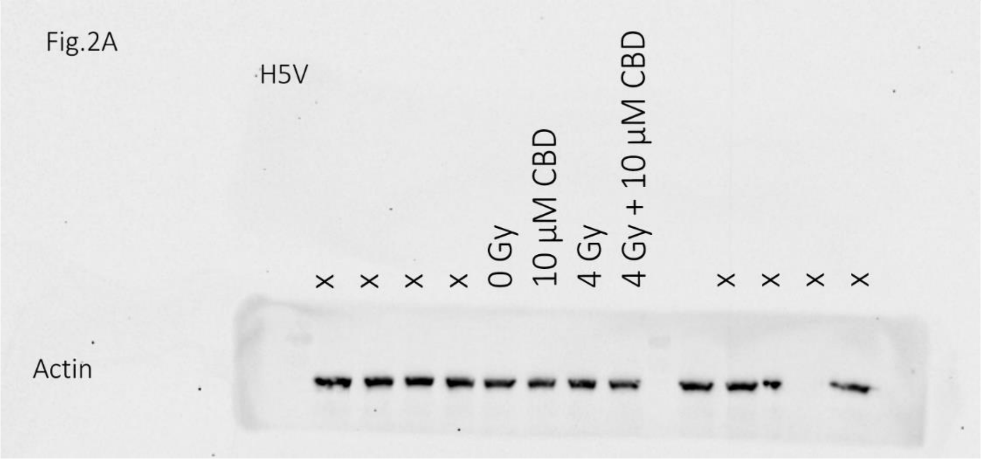

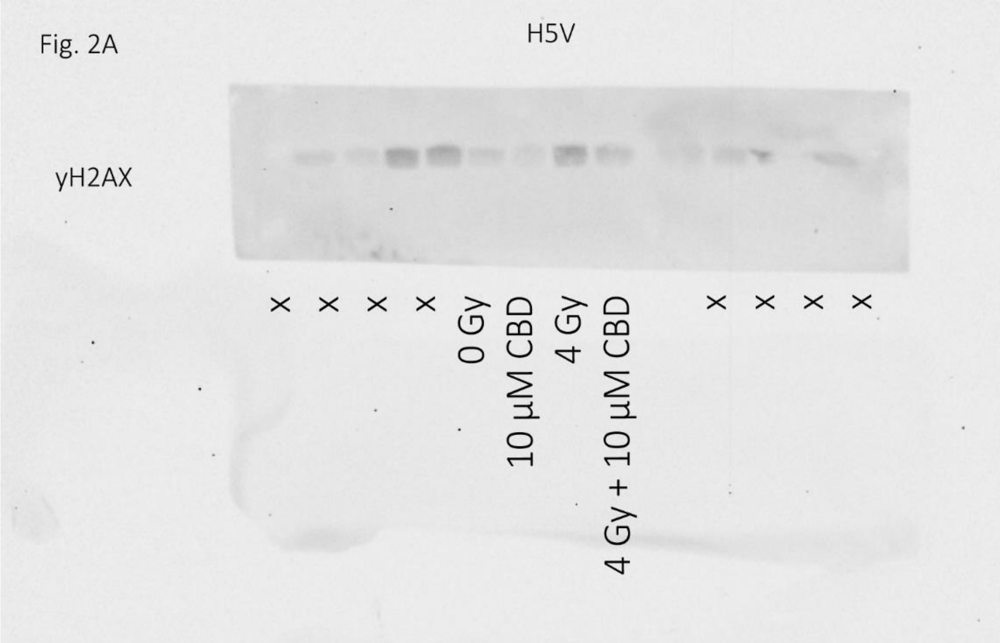

Figure S1. Annotated western blot images of figure 2A (n=1-3); intensity ratios (relative to β-actin) are given in supplementary table 2.

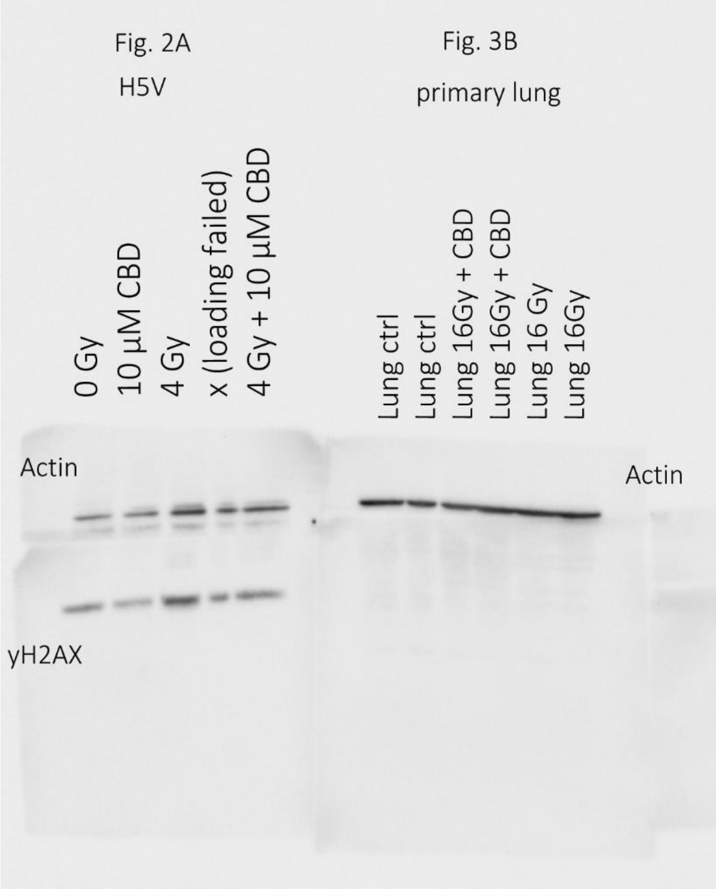

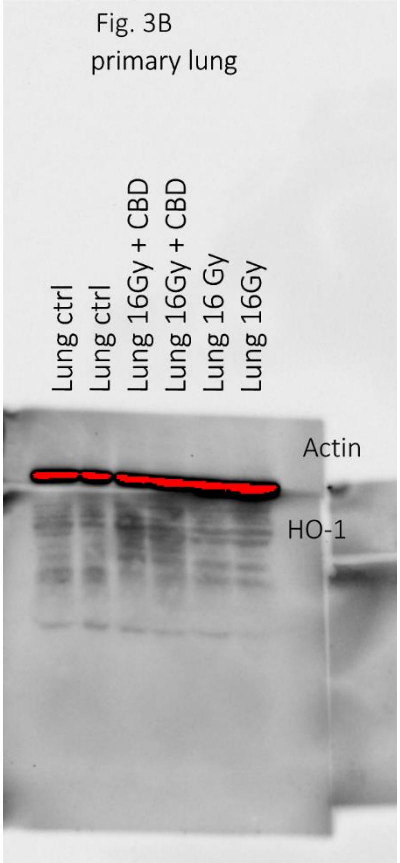

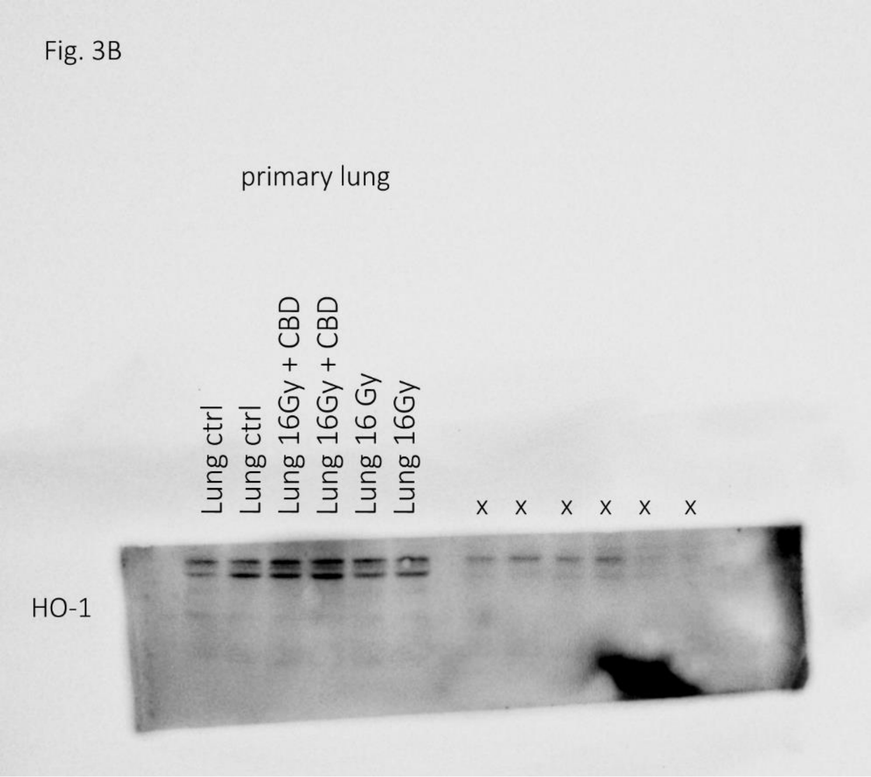

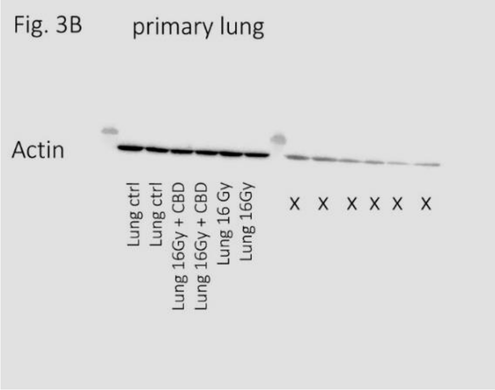

Figure S2. Annotated western blot images of figure 2A (n4) and figure 3B; HO-1 band intensity was measured at higher contrast due to weak bands; intensity ratios (relative to β-actin and normalized to control) are given in supplementary tables 2 and 3.

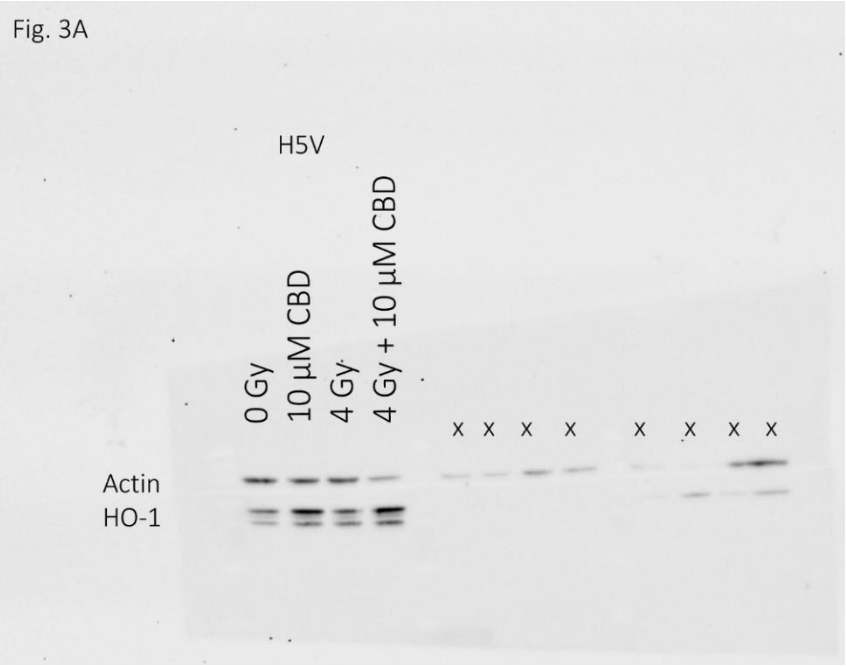

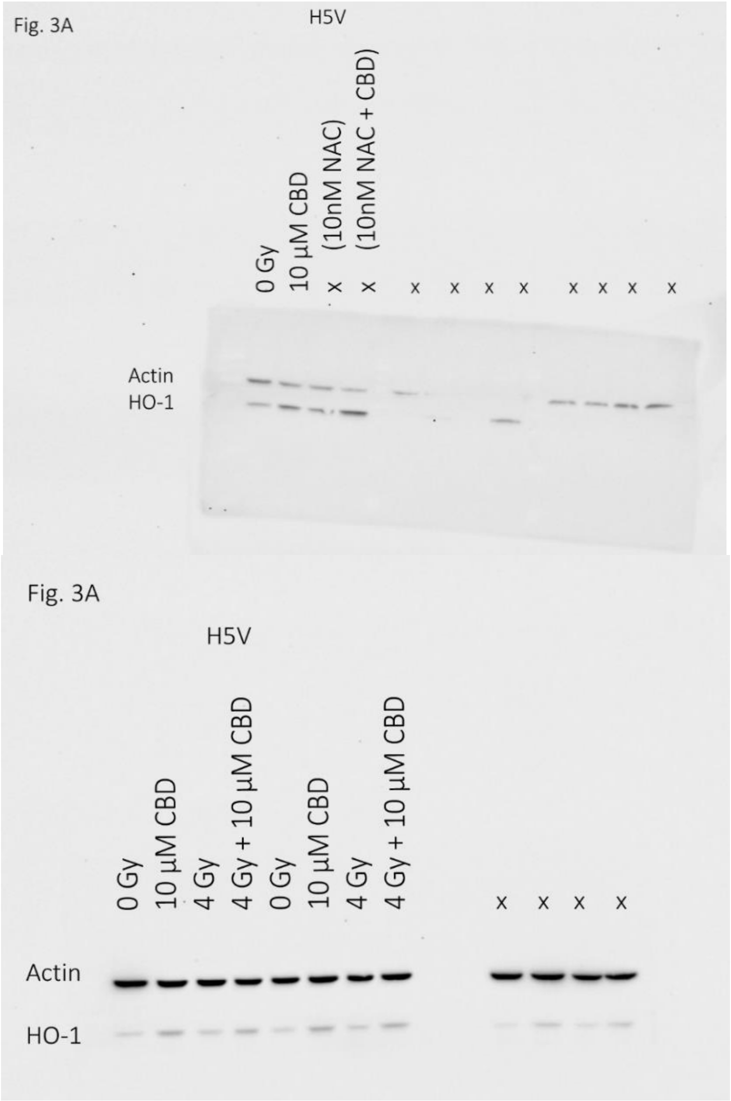

Figure S3. Annotated western blot images of figure 3A and appendix A3; intensity ratios (relative to β-actin and normalized to control) are given in supplementary table 3.
